# Décompression chirurgicale du syndrome de défilé thoraco-brachial

**DOI:** 10.11604/pamj.2014.19.77.4029

**Published:** 2014-09-24

**Authors:** Loubet Unyendje Lukulunga, Abdou Kadri Moussa, Mustapha Mahfoud, Farid Ismael, Mohamed Saleh Berrada, Moradh El Yaacoubi

**Affiliations:** 1 Orthopedic Department, Faculty of Medicine and Pharmacy, Mohammed V University, Ibn Sina hospital, Rabat, Morocco

**Keywords:** Décompression chirurgicale, syndrome de défilé thoraco-brachial, malformations osseuses, Surgical decompression, thoracic outlet syndrome, bone malformations

## Abstract

Le syndrome de défilé thoraco-brachial est une pathologie souvent méconnue à cause de diagnostic difficile par manque des signes pathognomoniques conduisant souvent à des errances. Les manifestations cliniques dépendent selon qu'il s'agit d'une compression nerveuse, vasculaire ou vasculo-nerveuse. Le but de cette étude est de décrire certains aspects cliniques particuliers et évaluer le résultat fonctionnel après la décompression chirurgicale du paquet vasculo-nerveux. Notre étude rétrospective a porté sur l'analyse des données cliniques, radiologiques, IRM et EMG sur les patients opérés entre janvier 2010 et juillet 2013 du syndrome de défilé thoraco-brachial dans le service de traumatologie orthopédie de l'hôpital Ibn Sina de Rabat. 15 cas ont été colligés: 12 cas post traumatiques (fracture de la clavicule) et 3 cas d'origines congénitales, dont l’âge moyen était 35 ans (20 à 50 ans) avec 9 femmes et 6 hommes. A la fin du traitement, le score de Dash est passé de 109 (46% Normal=0) à 70 (20%), et le stress test de Roos était de 70/100 à 80/100. Le résultat était excellent dans 12 cas soit (80%) et moins bon dans dans 3 cas (20%). En définitive, la résection de malformations osseuses, l'excision des brides et la neurolyse du plexus brachial suivie de la rééducation a donné une bonne évolution fonctionnelle.

## Introduction

Le syndrome de défilé thoraco-brachial décrit par COOPER en 1821 pour la première fois pour une compression vasculaire par une cote cervicale [1] c'est l'ensemble des manifestations cliniques liées à la compression intermittente ou permanente du tronc des plexus brachial, de l'artère ou de la veine sous-clavière lors de la traversée cervico-thoraco-brachiale dans un des compartiments. Six zones constituent cette traverséé cervico- thoraco-brachiale: il s'agit de défilé appareil suspenseur de la plèvre, défilé inter-scalenique, défilé pré-scalenique, le canal costo-claviculaire, le tunnel sous-pectoral et le billot huméral. Le syndrome de défilé thoraco-brachial est une pathologie souvent méconnue et sa fréquence est de 0.3 à 0.7% [2] à cause de diagnostic difficile par manque des signes pathognomoniques qui conduisent souvent à des errances malgré les multiples manœuvres d'Adson, de Wright, d'Eden et le test de Roos sans qu'ils ne soient spécifiques pour mieux décrire les symptômes artériels, veineux et neurologiques. Ses étiologies sont nombreuses à type d'anomalies congénitales musculo-ligamentaires et osseuses qui constituent 1% mais qui ne sont pathogènes qu’à 10% de cas [3]. Les manifestations cliniques dépendent selon qu'il s'agit d'une compression nerveuse, vasculaire ou vasculo-nerveuse. Jusqu'alors ce syndrome constitue un challenge pour le chirurgien en raison de son diagnostic difficile et sa complexité anatomopathologique. Actuellement avec le développement de l'imagerie, cette fréquence devient relativement élevée: 1 à 2% de la population. L’ absence de codification thérapeutique fait que son traitement ne puisse bénéficier de l'accumulation de compétences en traumato-orthopédie, chirurgie vasculaire, thoracique. Notre objectif est de décrire certains aspects cliniques particuliers et d’évaluer le résultat fonctionnel après la décompression chirurgicale du paquet vasculo-nerveux.

## Méthodes

**La série:** deux catégories selon leur étiologies, constituées des 15 patients ont été opérés dans le notre service de traumatologie pour syndrome de défilé thoraco-brachial: il s'agit de 12 cas post traumatiques de fracture de la clavicule et 3 cas d'origine congénitale, âgés 20 à 50 ans dont 9 femmes et 6 hommes. Les formes congénitales étaient bilatérales par méga-apophyse transverse C7 et côte surnuméraire associé à des brides fibreuses. Les critères d'inclusion étaient la présence des signes neurologiques ou vasculaires du syndrome de défilé. L’évaluation comportait un examen clinique utilisant le test de tunnel, le test de stress de Roos, la manœuvre de Wright et le questionnaire de Dash. Nous avons réalisé aussi un bilan de l'articulation gléno-humérale, de la coiffe des rotateurs et la mobilité active du rachis cervical.

**Figure 1:**
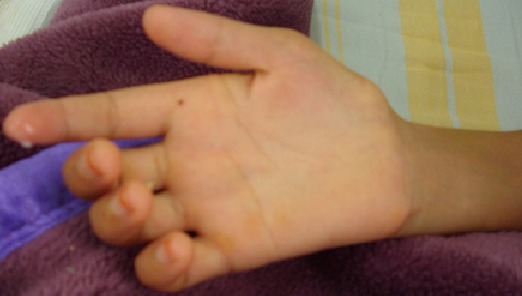
Aspect clinique du syndrome de la traverse thoraco-brachiale

**Signes cliniques:** les signes cliniques étaient dominés par paresthésies, sensation du bras mort, amyotrophie de l’éminence thénar et hypothénar (Figure 1). Les tests cliniques dynamiques de Roos et de chandelier étaient fiables pour l'atteinte neurologique.

**Figure 2:**
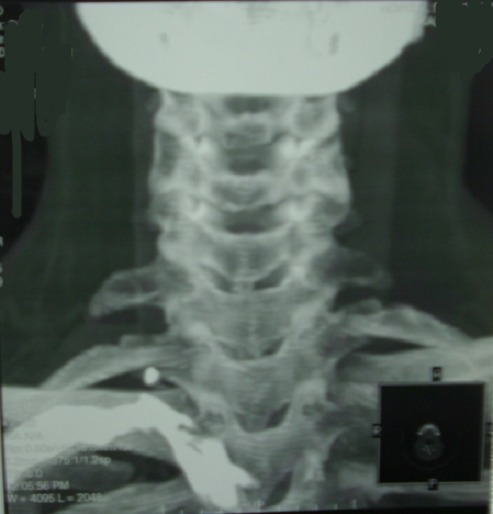
Radiographies de face objectivant la côte surnuméraire chez une patiente de 24 ans

**Imageries:** les radiolographies standards de face, profil, et de ^3^/_4_ du rachis cervical, de la clavicule, et du thorax complétées par le scanner avaient été demandées dans tous les cas et avaient objectivées les signes de fracture et de pseudarthrose ou de cal vicieux de la clavicule dans 12 cas et dans 3 cas la présence de méga-apophyse du C7 et de côte surnuméraire (Figure 2); -l'IRM à la recherche des anomalies musculo-ligamentaires; - l’électromyogramme de détection avait objectivé une atteinte plexi que du C8 à D1 dans les malformations congénitales; - l’écho-doppler étaient demandé systématiquement dans tous les cas à la recherche d'une sténose permanente ou anévrisme de l'artère sous Clavière et apprécier l’état du lit d'aval pour le diagnostic topographique; -l'angio- scanner avec reconstruction tridimensionnelle en 3D étaient demandées en position indifférente donne une bonne définition de l'atteinte vasculaire et de ses rapports osseux tout en précisant le siège de conflit.cas d'anomalies à l’écho-doppler. La compression était haute et avait concerné le plexus brachial et l'artère sous Clavière; -l'artériographie ou phlébographie ne sont demandées que lorsque les examens précédents sont défaillants.

**Etiologies:** 2 groupes d’étiologies ont constitué notre série parmi tant d'autres: 12 patients avec pathologies claviculaires post traumatiques et 3 patients avec une association de malformations congénitales faites de méga-apophyse, côte surnuméraire, et des brides fibreuses.

**Figure 3:**
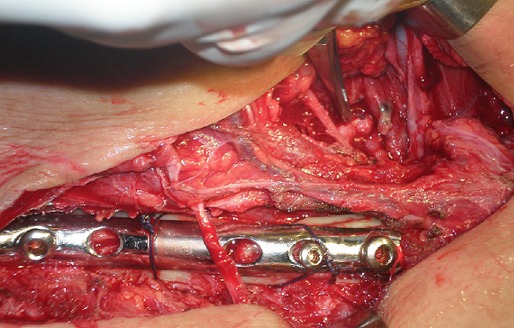
Ostéosynthèse de la clavicule après libération du paquet vasculo-nerveux chez un patient de patient de 50 ans sur fracture de la clavicule

**Figure 4:**
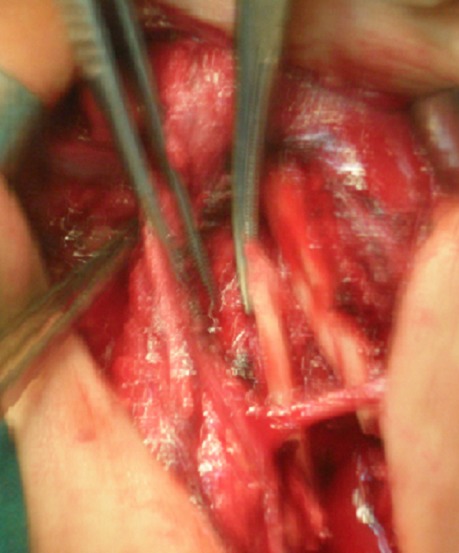
Libération du paquet vasculo-nerveux par la voie sus claviculaire

**Techniques chirurgicales:** tous nos patients ont été opérés par la voie sus claviculaire: les 12 patients présentant une compression vasculo-nerveuse sur fracture de clavicule avaient bénéficié de l'ostéosynthèse de la clavicule par plaque après libération du paquet vasculo-nerveux (Figure 3). Le deuxième groupe constitué de 3 cas de patients présentant une compression par des anomalies congénitales (méga-cervicale de C7, côte surnuméraire, associé à des brides fibreuses) ont été opérés avec les chirurgiens vasculaires par la même voie sus claviculaire et on a procédé à la résection de C7, de la côte surnuméraire, l'excision des brides et la neurolyse du plexus (Figure 4). Une reprise chirurgicale dans un cas opérés par la voie sus et sous claviculaire selon Cormier était utilisée pour reséquer la première côte. La rééducation utilisant le protocole de Peet modifié avec une fréquence de 3 à 5 fois/semaine pendant 8 semaines

## Résultats

A la fin du traitement, le score de Dash est passé de 109(46% Normal=0) à 70 (20%), et le stress test de Roos aussi était passé de 70/100 à 80/100. 10 patients sur 12 opérés fracture de la clavicule soit (83.3%) le résultat était excellent et moins bon dans dans 2 cas (12.7%). Les 3 patients opérés pour malformation congénitale, le résultat était bon à 67% et la récidive était observée dans 1 cas sur 3 soit 33%, nécessitant une reprise chirurgicale par la voie sus et sous claviculaire selon Cormier permettant de reséquer la première cote et les muscles scalènes.

## Discussion

Le syndrome de défilé thoraco-brachial est une pathologie souvent méconnue. La compression peut se situer dans un des compartiments que constituent cette traversée cervico- thoraco-brachiale. Chez 12 patients soit 80%, d’étiologie traumatique de la clavicule, la compression a existé au niveau de la pince costoclaviculaire [4] qui est limitée par la clavicule en haut, le muscle subclavier en avant, la 1^ère^ et la 2^ème^ côtes en bas et en arrière, à ce niveau, la veine subclaviere est en avant de l'artère et le plexus brachial en postérieur; tout mouvement d'hyper abduction du bras entraine un rétrécissement physiologique [5]. Cornelis avait trouvé dans une série de 56 cas; 18% des patients avec compression au niveau de la pince costoclaviculaire et dans 49% au niveau du triangle intercostoscalenique [6]. Pour les formes congétales, dans 20% le triangle intercostoscalenique était impliqué. Ce triangle est limité par le muscle scalène antérieur en avant, le bord supérieur de la première côte en bas, les muscles scalène moyen et postérieur en arrière. Il contient l'artère subclavière et le plexus brachial alors que la veine subclavière chemine dans l'espace préscalenique. Ce qui justifie la prédominance de signes neurologiques et artériels. Aucun cas n'a été trouvé au niveau du tunnel sous coracoïde situé en arrière du muscle petit pectoral, en avant du muscle subscapulaire et en dessous du processus coracoïde pouvant être aussi le siège de compression.

Dans les formes congétales la prédominance était féminine 66.6% avec manifestations neurologiques. Cette prédominance a été décrit dans la littérature [7]; Ceci s'explique par le fait que l'orientation de la première cote chez la femme est plus verticale et la ventilation plutôt thoracique est majorée par l'imprégnation oestroprogestative responsable d'une laxité musculo-ligamentaire [8]. Les malformations étaient bilatérales à 100% et la symptomatologie était plus marquée à droite qu’à gauche dans notre série. Ce qui est confirmé dans la littérature. Le manque des signes pathognomoniques malgré les multiples manœuvres d'Adson, de Wright, d'Eden et le test de Roos sans qu'ils ne soient spécifiques pour mieux décrire les symptômes artériels, veineux et neurologiques rend le diagnostic difficile. Mais Maisoneuve et Hachulla trouvent la manœuvre de Chandellier et le test de Roos plus spécifique que sensible [9, 10]. Les formes neurologiques pures étaient plus fréquentes et le niveau d'atteinte était basse entre les racines de C8 et D1. Les formes hautes sont rares moins de 5% de cas et touchent C5, C6, C7 [11]; Wood retrouve 44% de formes étagées [12]. Narakas en retrouve 32.5%. Ces compressions peuvent se trouver dans les territoires de nerfs médian au canal carpien, du nerf ulnaire au coude, et du nerf radial au court supinateur. Coote en 1861 effectua la première résection d'une cote cervicale pour compression vasculaire. Lorsqu'il s'agit des pathologies acquises de la clavicule, le diagnostic et le traitement sont faciles et nécessitent une libération de paquet vasculo-nerveux et une fixation de la clavicule par une plaque, 12 cas de notre série soit 80% avaient bénéficié de ce traitement. Mais en cas de malformation congénitale, les lésions sont complexes et associées qui rendent le diagnostic difficile et l'absence d'un consensus du traitement avec un taux très élevé des complications [13].

Notre voie d'abord était sus claviculaire dans tous le cas et nous avons obtenu 33% des complications qui seraient dues à l'insuffisance de la résection des scalènes. Maxwell et al [14] avaient obtenu 86.5% de disparation de symptômes par la seule résection de la première côte par voie sus claviculaire dans une série de 126 interventions. Ce que rejette Merle [15] qui estime que la morphologie de certains patients rend impossible la résection de l'arc antérieur de la première côte par la seule voie sus-claviculaire et que toute tentative aveugle risquerait de blesser la veine sous-clavière. Narakas [16] rapporte que la seule désinsertion des scalènes ne suffit pas à garantir un bon résultat et une résection sous périostée de la première cote peut induire sa reconstitution et les tissus fibreux qui se développent à partir du scalène antérieur et moyen créent à nouveau un véritable étranglement du plexus brachial. La voie axillaire de Roos ne met pas sans complications (10 à 20%), mais Roos lui-même dans sa série revendique d'excellents résultats (92%) et Merle sur 38 patients opérés par cette voie de Roos pour ablation de la première cote et neurolyse du paquet vasculo-nerveux avait obtenu 28 cas (73.6%) des complications ou insuffisances techniques. Lorsqu'il s'agit de forme vasculaire ou vasculo-nerveux, il admet la voie de Roos pour reséquer la première côte, et si la forme est neurologique pure il préfère la voie sus-claviculaire enfin de réaliser une scalénectomie, résection des structures ligamentaires et ablation d'une éventuelle côte cervicale. Mais pour traiter 4 des 6 étiologies Poitevin [17] utilise la voie sus et sous claviculaire de cormier [18], qui jusqu'alors n'a pas des complications décrites. Nous avons obtenu 33% de récidives qui avait nécessité une reprise chirurgicale par la voie sus et sous claviculaire selon Cormier permettant de reséquer la première cote et les muscles scalènes; les raisons étaient l'insuffisance technique et la fibrose cicatricielle qui avait cravaté le paquet vasculo-nerveux tel que le souligne Merle.

Certains auteurs préfèrent un traitement médical avec la kinésithérapie en première intention et la chirurgie n'interviendrait qu'en cas des formes aigues et invalidantes. Peet [18] en 1956 proposa un protocole basé sur la physiothérapie par correction gymnique de la posture et un renforcement des élévateurs qui permettait d'obtenir 70% d'amélioration. Mais ces protocoles de rééducation bien que donnant des résultats satisfaisants sont d'après leurs auteurs responsables des résultats insuffisants voir même d'aggravation des signes nécessitant la chirurgie [19].

## Conclusion

La décompression chirurgicale du syndrome de défilé thoraco-brachial par voie sus claviculaire suivie de la rééducation constitue un moyen thérapeutique qui améliore la disparition des symptômes. L'absence de codification thérapeutique malgré l'apport de l'imagerie fait que son traitement reste encore un challenge pour le chirurgien suite à sa complexité anatomique et ses associations lésionnelles qui quelles que soient la technique et la voie utilisée. En plus la dissection des muscles scalènes ainsi que de malformations osseuses et la fibrose cicatricielle ne seraient pas sans complications.
